# Multidrug-Resistant *Bacteroides fragilis* — Seattle, Washington, 2013

**Published:** 2013-08-30

**Authors:** Aley Kalapila, Steven Pergam, Paul Pottinger, Susan Butler-Wu, Estella Whimbey, Jeffrey Duchin

**Affiliations:** Univ of Washington Medical Center, Seattle; Public Health-Seattle & King County and Univ of Washington Medical Center, Seattle

The *Bacteroides fragilis* group consists of species of obligate anaerobic bacteria that inhabit the human gut. They are among the leading pathogens isolated in the setting of intra-abdominal infections. *B. fragilis* strains, especially in the United States, are virtually always susceptible to metronidazole, carbapenems, and beta-lactam antibiotics (1). Although isolated cases of resistance to single agents have been reported, multidrug-resistant (MDR) *B. fragilis* strains are exceptionally rare (1,2). In May 2013, an MDR *B. fragilis* strain was isolated from the bloodstream and intra-abdominal abscesses of a patient who had recently received health care in India. This is only the second published case of MDR *B. fragilis* in the United States. This report summarizes the case and highlights the need for awareness of multidrug-resistant organisms (MDROs) in returning travelers who have received inpatient medical care outside the United States, both for timely implementation of proper infection control measures and to ensure administration of appropriate antimicrobials.

## Case Report

A U.S.-born man aged 70–79 years, with past medical history notable only for benign prostatic hyperplasia, traveled to India for pleasure, arriving November 7, 2012. After traveling in India for 1 month, he developed progressive abdominal pain and sought medical attention at a hospital in Jaipur on December 11, 2012. During a 4-day hospitalization, he underwent colonoscopy with biopsy of a suspicious mass and was found to have a well-differentiated adenocarcinoma of the colon. Computerized tomography (CT) of the abdomen demonstrated multiple large liver lesions as well as pericolonic lymphadenopathy suggesting metastatic cancer. During his admission, he received 1 unit of packed red blood cells and several doses of unspecified intravenous antibiotics. He was advised to undergo surgical resection of his cecal mass but decided to travel to New Delhi to seek a second medical opinion. There, he was hospitalized during January 5–9. Based on limited records from that admission, it does not appear that antibiotics were administered at that time. The patient then returned to the United States and was evaluated at a cancer center in Seattle, Washington, where he received five cycles of chemotherapy as an outpatient in early February 2013. He received 3 days of oral levofloxacin for a brief episode of neutropenia during chemotherapy.

In May 2013, after chemotherapy, the patient was admitted to the University of Washington Medical Center for a complex tumor resection. He received single doses of preoperative cefazolin and metronidazole. On postoperative day 4 he developed leukocytosis with a maximum white blood cell count of 25,000/*μ*L. Blood cultures were obtained but yielded no growth. A CT scan of the abdomen revealed multiple fluid collections suggesting abscesses. Vancomycin and piperacillin/tazobactam were initiated, and the patient underwent radiographically guided percutaneous drainage. The fluid grew a pan-susceptible *Escherichia coli,* and antibiotics were narrowed to ceftriaxone. The leukocyte count improved initially, but then increased again several days later. Repeat blood cultures drawn through a central catheter showed anaerobic gram-negative rods, and piperacillin/tazobactam coverage was restarted. Follow-up blood cultures drawn 2 days later demonstrated no growth. A repeat CT scan for persistent fever, 10 days after drain placement, demonstrated a ring-enhancing fluid collection in the abdomen and right flank and pelvic fluid collections. Vancomycin was added to the patient’s antimicrobial regimen, and an additional percutaneous drain was placed. Fluid was sent immediately for microbiologic testing. Gram stain of the fluid revealed 4+ polymorphonuclear cells and 3+ gram-negative bacilli, with a pure culture of anaerobic gram-negative rods isolated in culture.

Both blood culture and abdominal fluid culture isolates were identified as *B. fragilis*. Both isolates demonstrated high levels of resistance by epsilometer test (E-test) to multiple antibiotics, including metronidazole, imipenem, piperacillin/tazobactam, and clindamycin. Resistance to cefotetan, ampicillin/sulbactam, and moxifloxacin also was observed.

The patient was placed under contact precautions (3), and antimicrobials were changed temporarily to imipenem and metronidazole while additional susceptibilities were performed, including to tigecycline, minocycline, and linezolid. Cultures from additional percutaneous drains placed in the intra-abdominal fluid collections also grew MDR *B. fragilis*. Species identification was confirmed as *Bacteroides fragilis ssp. fragilis* by biochemical testing, mass spectrometry, and molecular sequencing. Microbiologic testing for the blood isolate demonstrated susceptibility to minocycline, linezolid, and tigecycline. Based on a published report of successful use of linezolid for the treatment of an intra-abdominal infection with MDR *B. fragilis* (4), the patient’s regimen was changed to linezolid and ertapenem to treat this organism and other probable gram-negative rods associated with his intra-abdominal abscesses.

The patient remained afebrile with negative subsequent blood cultures. He was discharged from the hospital on an outpatient regimen of oral linezolid and parenteral ertapenem. His abdominal abscesses gradually resolved, and his antibiotics were discontinued after approximately 4 weeks of treatment. He remains under strict contact precautions during all inpatient and outpatient health-care treatments.

### Editorial Note

A national survey of the susceptibility of *B. fragilis* analyzed approximately 6,000 isolates from 13 medical centers during 1981–2007 for antimicrobial resistance. The survey noted <1% resistance in the *B. fragilis* group to imipenem/cilastin, and only three isolates demonstrated resistance to metronidazole (1). In Europe, resistance to imipenem/cilastin or metronidazole has been reported in only 1%–2% of isolates (2). There are different mechanisms of resistance to these antimicrobial agents. The *cfiA* gene, which is typically chromosomal, encodes for metallo-beta lactamases that confer carbapenem resistance (5). Metronidazole resistance, however, has been reported as typically caused by *nim* genes that are either located on plasmids or on the chromosome (5). *B. fragilis* isolates that simultaneously express multiple mechanisms of resistance to different antibiotic classes are exceedingly rare, with only a handful of case reports worldwide. Testing for molecular mechanisms of resistance to metronidazole and carbapenems in the described patient’s isolate are under way.

In this report, the patient received short courses of six antibiotics during his admission to the University of Washington Medical Center, which might have played a role in the genesis of his MDR *B. fragilis*. However, before that admission, he had traveled to India, where he was hospitalized twice and underwent an invasive procedure. Recently, cases of carbapenem-resistant Enterobacteriaceae (CRE) have been associated with inpatient admissions in medical facilities outside of the United States, including in hospitals in India (6). Although metronidazole resistance in *B. fragilis* has been reported in India (7), it is extremely rare throughout the world. This is only the second case of MDR *B. fragilis* infection reported in a U.S. hospital with resistance to both carbapenems and metronidazole. The first U.S. case, reported in 2011, was in a U.S. Army soldier with MDR *B. fragilis* isolated from blood and tissue following an injury sustained in Afghanistan (8).

Recent interest in infection control measures surrounding MDROs focuses on CRE infections in returning travelers, especially those coming from the Indian subcontinent. The case described in this report, as well as the previous MDR *B. fragilis* case in the United States (8), suggests that other MDR bacteria that pose a potential public health threat could be associated with recent international travel. The most recent Clinical and Laboratory Standards Institute guidelines do not recommend routine susceptibility testing of anaerobes except in the case of serious infections or failure of standard antimicrobial therapies (9). Therefore, heightened vigilance is needed for the possibility of MDROs in patients who have received health care outside the United States. Most importantly, this case reinforces the importance of identifying patients at risk for MDROs and implementing early empiric contact precautions and other infection control measures for patients who received inpatient medical treatment outside of the United States (3).

What is already known on this topic?

*Bacteroides fragilis* are anaerobic bacteria found in the human gastrointestinal tract and often cause intra-abdominal infections. They are typically susceptible to a variety of antimicrobials, including carbapenems and metronidazole. Resistance to these antibiotic classes, particularly in combination, is extremely rare.

What is added by this report?

A *B. fragilis* strain that was highly resistant to multiple antibiotics, including carbapenems and metronidazole, was isolated from a patient with an intra-abdominal abscess who had been hospitalized recently in India. This is the second reported case in the United States of a *B. fragilis* stain with this unusual resistance pattern.

What are the implications for public health practice?

Clinicians, who are becoming increasingly aware of carbapenem-resistant Enterobacteriaceae, should also be vigilant to the possibility of multidrug-resistant bacteria such as *B. fragilis* when caring for patients who have received inpatient medical care outside the United States. Such vigilance can be important for timely institution of infection control measures and selection of appropriate antibiotics.

Although *B. fragilis* has long been considered reliably susceptible to a number of broad-spectrum anti-anaerobic drugs (1), the case in this report and others like it (10) suggest clinicians should no longer rely on cumulative susceptibility data from surveys alone to direct treatment and should consider requesting susceptibility testing when treating serious infections caused by *B. fraglis*. Nonetheless, drainage of abscesses and surgical debridement of involved tissue remain the cornerstones for treating most anaerobic infections.

This case also suggests an expanding scope of multidrug resistance and the need for improved antibiotic stewardship. Similar to the Enterobacteriaceae, *B. fragilis* is a normal part of the human lower intestinal microbiota. Increasing clinical infections caused by MDRO strains of such disparate bacteria as Enterobacteriaceae and *B. fragilis* might be a sentinel for a larger expansion of resistance. Although antibiotics have saved countless lives and allowed modern medicine to advance rapidly, their use to treat infections is a global public health resource that needs to be carefully conserved, both in the United States and abroad.
